# Factors Influencing Social Isolation among Cancer Patients: A Systematic Review

**DOI:** 10.3390/healthcare12101042

**Published:** 2024-05-17

**Authors:** Can Wang, Xiaoke Qiu, Xueli Yang, Jiayu Mao, Qiuping Li

**Affiliations:** Wuxi School of Medicine, Jiangnan University, Wuxi 214122, China; 6232807028@stu.jiangnan.edu.cn (C.W.); 6232807022@stu.jiangnan.edu.cn (X.Q.); 6232807039@stu.jiangnan.edu.cn (X.Y.); 6232807048@stu.jiangnan.edu.cn (J.M.)

**Keywords:** cancer, social isolation, loneliness, influencing factor

## Abstract

(1) Background: Social isolation, which has numerous adverse effects on health status, is prevalent among cancer patients. This review proposes to identify the influencing factors of social isolation among cancer patients. (2) Methods: Articles published in English or Chinese from six electronic databases before December 2023 were identified via a systematic search. A manual search was also performed. (3) Results: Twenty-eight studies were identified in this systematic review. The factors associated with social isolation can be summarized into the following categories: demographic characteristics, having cancer, health status, coping, social support and social interaction. Despite the heterogeneity, 20 factors were significantly associated with social isolation, including age, gender, comorbidity burden, education level, residence, medical insurance, occupation status, personality, race, smoking status, having children, not living alone, household income level, marital status, the role of primary caregiver, physical health status, mental health status, social health status, coping styles, and the level of social support and social interaction. (4) Conclusions: The systematic review showed that cancer patients’ social isolation was influenced by their demographic characteristics, cancer-related factors, physical condition, psychological status, social health status, coping styles, and level of social support and social interaction. In addition, future group intervention could be considered to improve social isolation.

## 1. Introduction

According to 2020 statistics [1], approximately 19.3 million people worldwide are diagnosed with cancer each year. Projections indicated that by 2040, the global burden of cancer will reach 28.4 million cases, representing a significant increase of 47% compared to 2020 [1]. With the rapid improvement in medical technology, there are an ever-increasing number of cancer survivors [2]. However, patients undergoing cancer treatments suffer from many kinds of physical burden, as well as psychosocial stress, e.g., social isolation [3,4]. Therefore, attention should be paid to not only the survival rate of patients but also psychosocial aspects such as the social isolation of patients with cancer.

### 1.1. Defining the Incorporated Construct of Social Isolation

As initially proposed by Berkman and Syme in 1979, social isolation is defined as a state characterized by the absence of social networks and support [5]. Subsequently, Lien-Gieschen emphasized the subjective and perceived status of individual experiences caused by inadequate social support [6]. Fine and colleagues also argued that subjective emotional experiences such as loneliness should also be included in the concept of social isolation [7]. The term social isolation has been utilized in the academic literature across multiple disciplines including sociology, medicine, and nursing [4]. However, due to the different research objectives and research fields, a consensus regarding its definition has not yet been reached between and even within various disciplines. Researchers have defined the concept of social isolation in various ways [4]. While some viewed social isolation as a unidimensional concept referring solely to the lack of social integration (an objective measure) [8,9], others considered it as multi-dimensional, encompassing both objective and perceived aspects of isolation [10,11]. In this study, we adopt the latter perspective.

It is worth noting that loneliness and social isolation are often used interchangeably within the academic literature [12,13]. However, they differ slightly in their definitions [14]. Loneliness is usually portrayed as “a negative emotional state resulting from the dissatisfaction with unmet needs or expectations in actual social relationships” [15]. What is more, Weiner differentiated emotional loneliness, referring to the perceived status occurring with the absence of desired companionship, as well as from social loneliness, an emotional experience resulting from the lack of a wider social network and social interaction [16]. Emotional loneliness is often correlated with the absence of intimate relationships, while social loneliness is often associated with the loss of social networks [16]. According to these related definitions, loneliness is considered to be the subjective component of social isolation [17,18]. Therefore, our study will review studies related to both social isolation and loneliness.

### 1.2. The Adverse Effects of Social Isolation or Loneliness

With the growing interest in investigating social isolation in recent years, researchers have confirmed that social isolation, or loneliness, has numerous adverse effects on the health status of cancer patients [19,20]. Studies have found that social isolation or loneliness was a risk factor for the following physical and mental statuses: malnutrition, new-onset chronic conditions, risky health behaviors, anxiety, constructive coping, depression, illness acceptance, and suicidal ideation [21,22,23,24]. Additionally, there was evidence that social isolation and loneliness were even related to all-cause and cancer mortality [25].

The high prevalence of social isolation among cancer patients [26], combined with compelling evidence demonstrating its detrimental impact on physical and mental well-being [21,23], underscores the urgency of addressing social isolation as a critical health concern. Therefore, it is vital to identify the influencing factors of social isolation among cancer patients and develop corresponding intervention programs. 

### 1.3. The Potential Factors Influencing Social Isolation or Loneliness

Personality is a dynamic organization within an individual, consisting of psychophysical systems that give rise to characteristic patterns of behavior, thoughts, and emotions [27]. Different individuals with various personalities may adopt different coping styles when dealing with stress-causing issues such as having cancer. Therefore, the effects of cancer vary from person to person, leading to differences in the level of social isolation due to their personality traits. For instance, role function is a measure of quality of life [28], which is related to whether an individual is unable to perform the duties required by their social role because of health problems. The assessment of the role function of cancer patients can help us to understand the impact of their health status on their daily life and social participation. Role function may be influenced by their personalities [29], leading to a higher or lower score for social isolation. 

The concept of the patient role was introduced by American sociologist Parsons [30], and role adaptation entails aligning one’s psychology and behavior with the requirements of being a patient, including facing reality objectively, acknowledging illness, and actively seeking treatment measures [30]. For example, during the diagnosis and treatment of cancer, individuals need to transition from other societal roles to the role of a cancer patient. However, this process may present challenges in successfully completing the role transition due to maladaptation issues, leading to adverse effects on cancer patients.

Previous systematic reviews on the influencing factors of social isolation or loneliness among cancer patients focused on different targeted populations with cancer, e.g., Pilleron and colleagues focused on older cancer patients [31], while Fox et al. concentrated on young adult cancer survivors aged 18–39 years old [20] and Pahl and colleagues centered on adolescent cancer patients aged 10–21 years and survivors of childhood cancer diagnosed with cancer prior to age 21 [32]. 

A meta-analysis published in 2014 reviewed quantitative studies and explored the influencing factors of loneliness and objective social isolation among cancer patients, including both cancer-related factors and non-cancer-related factors [14]. Deckx and colleagues reported that the type of cancer and treatment and stage of disease were not associated with loneliness, while time since cancer diagnosis, marital status, and social support were correlated with loneliness [14].

In the past decade, reviews of adult cancer patients related to the factors affecting social isolation, which include both objective and subjective aspects, have been lacking. Therefore, this paper, based on the review of factors influencing loneliness in cancer patients published in 2014, searched quantitative articles published in English before 24 September 2013 [14]; by verifying and analyzing the influencing factors of social isolation in cancer patients without the limitation of study design, we provide some suggestions for improving social isolation in patients with cancer.

## 2. Materials and Methods

### 2.1. Search Methods for Eligible Articles

This review’s methodology satisfied the PRISMA (Preferred Reporting Items for Systematic Reviews and Meta-Analyses) guidelines [33]. The review was registered on the Open Science Framework (https://doi.org/10.17605/OSF.IO/M6AVU (accessed on 15 April 2024)). The Cumulative Index of Nursing and Allied Health Literature (CINAHL), the Cochrane Library, Embase, PsycINFO, PubMed, and the Chinese National Knowledge Infrastructure (CNKI) were retrieved for articles published in English or Chinese. While social isolation and loneliness are recognized as distinct concepts, the terms were often used interchangeably. Therefore, we retrieved all eligible articles published before December 2023 from the above databases and used the following free terms in the title, abstract, or subject: ‘Social isolation’ or ‘loneliness’ AND ‘Cancer’ or ‘Tumor’ or ‘Oncology’ or ‘Neoplasms’ or ‘Neoplasia’. In order to ensure the consistency of the search strategy of previous review [14], a free terms search was intended to be uniformly adopted in this survey. A manual search of references cited in the included articles was also conducted, and four additional articles were included.

### 2.2. Selection Criteria for Identifying Articles

The inclusion criteria for the literature were as follows: (1) Focused on adult patients with a pathological or clinical diagnosis of cancer (at an age equal to or more than 18 years old). (2) Quantitative studies were published after 24 September 2013 (a review with a similar purpose included all eligible quantitative studies published before 24 September 2013), and no time limits for qualitative or mixed method studies. (3) Studies were completely published in peer-reviewed journals in English or Chinese (no time limits for articles in Chinese).

The exclusion criteria for the literature included the following: (1) Not adult cancer patients. (2) Focused on loneliness related to specific circumstances (e.g., appearance concerns, dementia, having barriers in culture and language, accepting palliative care). (3) No primary qualitative or quantitative data. (4) Social isolation or loneliness was not measured via a validated scale in quantitative or mixed method studies. Studies that used single questions or directly asked about a person’s perceived level of loneliness were excluded. (5) Studies were reviews, meta-analyses, dissertations, commentaries, conference abstracts, or editorials.

### 2.3. Quality Assessment

The Mixed Methods Appraisal Tool (MMAT) was utilized for the assessment of the included studies [33]. The MMAT enables critical appraisal of diverse study designs, including qualitative, quantitative, and mixed methods studies. The quality assessments of each article using MMAT were conducted by two reviewers, engaging in discussions until a consensus was reached in the event of disagreements. 

### 2.4. Data Extraction and Synthesis

Data were extracted from the included articles by utilizing a pre-designed data-charting form that encompassed the following elements: author, year of publication, country, study objective, sample, instrument employed, key findings, and classifications of influencing factors.

As a study for decreasing loneliness, Stewart et al. proposed a conceptual framework [34], which assumed that stress (i.e., having cancer), health status (i.e., physical status, psychological status), coping, and social support would be affected and could be improved by group intervention. Moreover, these were proven to interact with each other. Therefore, guided by the conceptual framework, our study focused on analyzing and synthesizing the influencing factors of social isolation as five aspects, including demographic characteristics, having cancer, health status, coping, and social support and social interaction.

#### 2.4.1. Having Cancer

Obviously, being diagnosed with cancer is a stressful event. The cancer itself and the following treatments can give rise to certain complications for individuals with cancer [35]. Therefore, we introduced cancer-related factors. 

#### 2.4.2. Health Status

Health status mainly encompasses physical and psychological health status [36], with the World Health Organization emphasizing social well-being as an integral part of health [37]. Therefore, we aimed to explore the associations between social isolation and physical health status or psychological health status, as well as the relationship between social isolation and social health status such as role function. 

#### 2.4.3. Coping

Coping, defined by Folkman and Lazarus in 1986, refers to the cognitive and behavioral efforts a person makes to cope with the internal and external demands of interacting with the environment when judging that the interaction with the environment may burden themself or even exceed the resources at their disposal [38]. Coping style refers to the cognitive and behavioral style taken by individuals in the face of frustration and pressure [39], and it is an important intermediary factor in the process of psychological stress [40].

#### 2.4.4. Social Support and Social Interaction

Broad social support includes both structural social support and functional social support, while social support in the narrow sense means functional social support [41]. Structural social support refers to the number of people socializing, the number of diversities in networks, and the frequency of social interaction [41]. Functional social support is usually considered as perceived company, emotional support, and intangible social support [42]. Social interaction is the measure of structural social support [43], whereas social support, also called function social support, may be provided by social interaction [43]. What is more, social support and social interaction are protective factors of negative incidents [44,45]; thus, social support and social interaction represent one aspect among several influencing factors. There have been many other demonstrations of social support. For example, social support has been indicated to include both emotional social support and instrumental social support [46]. Emotional support encompasses comfort, encouragement, and empathy from others, as well as the provision of emotional security and trust. This form of support enables the recipient to feel emotionally bolstered and reassured in the face of adversity. Instrumental support involves practical assistance, resource sharing, and problem-solving support provided by others, including financial aid. This type of support can help individuals to more effectively alleviate the pressure and burden that they experience, thereby enabling them to better navigate difficulties and challenges [47].

## 3. Results

### 3.1. Process of Study Selection

**Figure 1:**
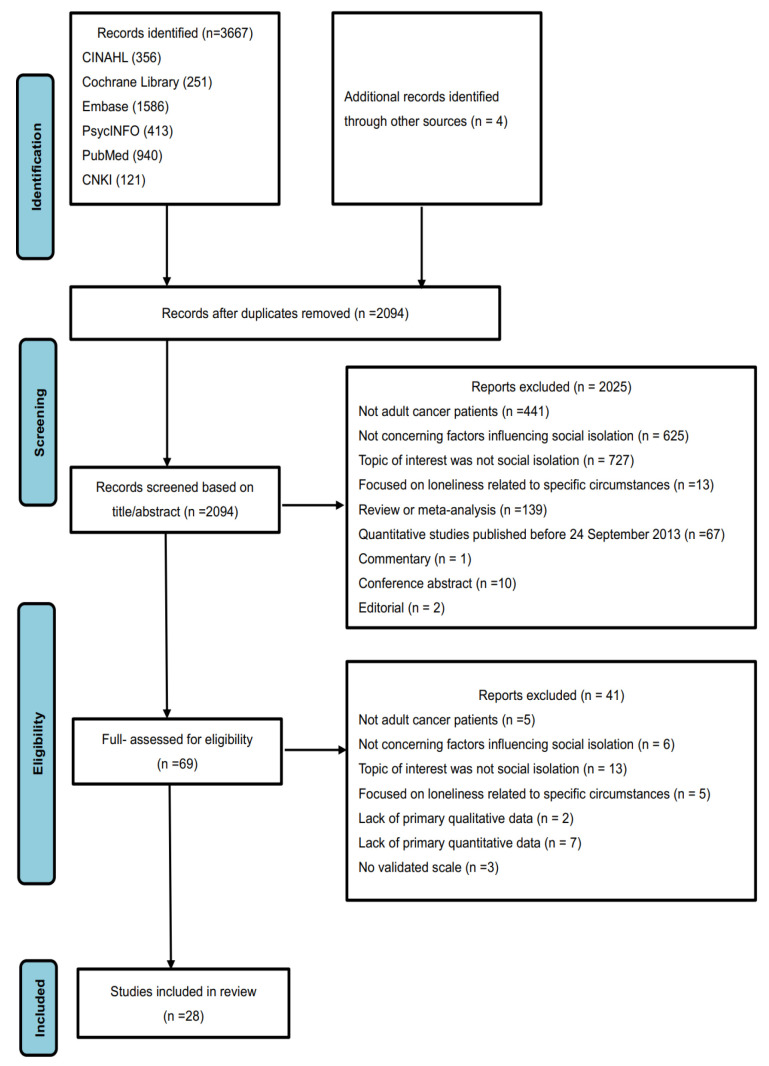
The flow diagram on identifying the literature.

A total of 3667 articles were identified from the Cumulative Index of Nursing and Allied Health Literature (CINAHL), the Cochrane Library, Embase, PsycINFO, PubMed, and the Chinese National Knowledge Infrastructure (CNKI). After removing 1577 duplicates, a further 2025 articles were excluded based on title and abstract screening, and an additional 41 articles were excluded after reading the full text. In total, 28 relevant articles were identified after critical appraisal. The reasons for study exclusion are presented in Figure 1.

### 3.2. Result of Quality Assessment

As Hong et al. (2018) discourage this, overall quality scores for the studies were not accumulated, but the methodological quality of the studies according to the MMAT was assessed [33]. For the general quality of the included 28 studies in the present review, only one study had two “Cannot tell” ratings, while 10 studies had one “No” or “Cannot tell” rating. Accordingly, it could be concluded that the general quality of these 28 studies was believed to be good. The results of the quality appraisal of the included studies are presented in Table 1.

### 3.3. Characteristics of Study

The 28 studies were conducted in China (*n* = 14), the USA (*n* = 9), Turkey (*n* = 3), Australia (*n* = 1), and Japan (*n* = 1). Regarding the study design, thre3e were qualitative studies, 24 were quantitative studies, and 1 was a mixed method study. Of the quantitative studies, one was prospective and the others were cross-sectional surveys. The study methods utilized in the qualitative studies encompassed open-ended interviews and semi-structured interviews. 

Additionally, of the 28 studies, 24 measured subjective social isolation, 1 assessed objective social isolation [22], and 3 measured both subjective social isolation and objective social isolation [10,48,67].

In terms of our careful statistics, the sample sizes of the included studies ranged from 13 to 104,640 patients, and study participants had been diagnosed with breast cancer, ovarian cancer, cervical cancer, prostate cancer, colorectal cancer, lung cancer, or mixed cancer. Among the 28 studies, 2 studies included individuals without a history of cancer. The details (study aims, samples, instrument used, key findings, and classifications of influencing factors) can be seen in Table 2.

### 3.4. Classification of Factors Associated with Social Isolation

The factors associated with social isolation among cancer patients are complex and, in addition to demographic characteristics, can be summarized into the following categories based on the conceptual framework [34]: having cancer, health status, coping, and social support and social interaction. The presentation of the survey results will be conducted mainly in alphabetical order of the relevant content.

#### 3.4.1. Demographic Characteristics

For the sake of comprehensiveness, in addition to the four aspects included in the framework [34], we also conducted an integrated analysis of the relationships between demographic characteristics and social isolation, which aimed to deepen our understanding of the factors contributing to social isolation. Among the 28 included studies, a total of 17 studies reported a significant association between social isolation and the demographic factors of cancer patients. In accordance with our analysis, we would discuss patient-related factors, including age, gender, comorbidity burden, education level, residence, medical insurance, personality, occupation status, race, and smoking status, as well as family-related factors such as family income level, having children, living situation, marital status, and the identity of the primary caregiver. 

Patient-related factors: Findings about the relationship between age and social isolation were inconsistent. Some studies confirmed that younger patients experienced greater feelings of isolation [55,61,67,70], while others certified that older patients had a greater score for subjective social isolation [57]. Similarly, studies regarding the relationship between gender and social isolation were inconclusive, with some findings showing that male patients felt more lonely [53] and others suggesting that female patients were more prone to subjective or objective social isolation [54,56,68,70,73]. As for the results reported by a recent work, the comorbidity burden also could affect patients’ loneliness to a certain extent [70]. For the education level, the results of five studies all indicated a negative correlation between education level and subjective or objective social isolation [53,56,65,67,68], demonstrating that higher education levels were associated with lower levels of subjective or objective social isolation. In addition, patients who lived in rural area [68], purchased the new rural medical insurance [68], were type D personality [57], and were unemployed or on sick leave [67] were more likely suffer from subjective social isolation. Specifically, non-Hispanic Black and non-Hispanic Asian participants showed decreased loneliness scores compared to non-Hispanic White patients in a prospective study [70]. Furthermore, two studies examined the relationship between smoking status and subjective social isolation, revealing that current smokers had higher levels of loneliness compared to never-smokers [59,70].

On a personal level, age and gender were both correlated with social isolation, but there has not yet been a consensus on the results of the relationships. Comorbidity burden, a lower education level, living in rural areas, purchasing new rural medical insurance, type D personality, unemployment or being on sick leave, being non-Hispanic White, and smoking were proven to be risk factors of social isolation. 

Family-related factors: The findings from three studies indicated a negative association between the level of household income and the degree of subjective social isolation [10,63,73], suggesting that the higher the level of family income, the lower the degree of subjective social isolation. In a cross-sectional survey of 90 breast cancer patients, Choi and Henneghan indicated that not having children may be a risk factor for increased levels of loneliness in patients [55]. The other study conducted on 100 older adults with cancer, showed that patients who lived alone had significantly higher loneliness scores than the others [73]. Additionally, results from seven studies showed that marital status might impact the social isolation of patients with cancer [53,57,59,66,67,71,73]. The results illustrated that married patients would experience lower feelings of isolation, while patients who were single would be more likely to experience social isolation. Apart from these, scholars estimated the influence of primary caregivers [69] and confirmed that enterostomy patients whose primary caregiver was their parent were more likely to suffer from subjective social isolation [69].

At the relational level, findings demonstrated that higher family income levels, having children, not living alone, and marital status were protective factors against social isolation, indicating that they were negatively correlated with the level of social isolation. Patients whose primary caregiver was their parent had higher scores of social isolation than other types of primary caregivers.

#### 3.4.2. Having Cancer

Next, we would introduce cancer-related factors, such as cancer type, treatment stage, etc. In total, 12 of the 28 studies analyzed the relationships between cancer-related factors and social isolation, including the type and stage of disease and treatment, hospitalization frequency, treatment-related time, and informed status.

According to our review, different cancer diagnoses and disease stages could influence the scores of social isolations. For example, one study on urological cancer patients demonstrated that bladder cancer patients had significantly higher scores of loneliness than prostate and renal cancer patients [71]; the other study on ovarian cancer revealed that patients with late-stage disease had the highest levels of feelings of isolation [57]. As demonstrated by five studies, treatment type and stage might impact the extent of social isolation. Three studies on colorectal cancer patients, breast cancer survivors, and urological cancer patients that depicted patients with temporary enterostomy [69], undergoing radical mastectomy [67] or accepting systemic treatment [71], respectively, had higher scores of subjective or objective social isolation. And the results of two studies revealed that patients in the treatment phase of surgery and chemotherapy were more likely to experience subjective or objective social isolation [10,68]. As shown in a study led by Kömürcü, the cycle of chemotherapy was positively correlated with loneliness [61]. Furthermore, the number of hospitalizations was illustrated to be positively related to the level of subjective social isolation [69]. Apart from that, treatment-related time such as postoperative time and time since the last treatment were estimated in Western and Eastern surveys. Two studies on breast cancer [67] and colorectal cancer [56], respectively, both indicated that postoperative time was negatively related to subjective or objective social isolation. However, the results of three studies on the relationship between time since the last treatment and the feelings of isolation were controversial [55,63,65]. In addition, results from two studies indicated that informed patients had greater feelings of isolation than ignorant patients [54,68]. 

In summary, despite the heterogeneity, cancer-related factors can be considered to impact cancer patients with social isolation via disparate paths.

#### 3.4.3. Health Status

For physical health status: Seven studies showed that patients’ physical status was greatly correlated with social isolation [48,49,51,53,54,59,72], demonstrating that patients with worse performance status would have more feelings of isolation. Some detailed influencing factors such as loss of appetite, nausea, vomiting, pain, shortness of breath, sleeplessness, and poor sleep quality could significantly affect the level of social isolation [53,54,72]. 

For psychological health status: Of the invested studies, 18 collected data on the psychological status of cancer patients. General mental status and specific psychological status such as anxiety and depression, fear of cancer recurrence, sadness, negative social expectation, self-perceived burden, stigma, self-esteem, and self-transcendence were proved to be significantly correlated with the level of social isolation.

According to five studies, poor general mental status was considered to be a significant influencing factor of subjective social isolation [48,51,54,60,62]: the poorer mental status, the higher the level of subjective social isolation. What is more, anxiety and depression were proven to be positively correlated with subjective or objective social isolation [22,51,64,66,70]. Other negative emotions, such as fear of cancer recurrence [72], negative social expectation [59,64], self-perceived burden [58], and stigma [59,69], were also positively correlated with subjective social isolation. What is more, findings showed that patients would feel alone in their awareness of mortality in a qualitative study [50]. Meanwhile, results indicated that positive psychological statuses including self-esteem [22] and self-transcendence [10] were negatively correlated with the score for subjective or total social isolation, demonstrating that improving the level of self-transcendence and self-esteem could possibly decrease the level of social isolation. 

For social health status: The role function was proven to be negatively correlated with loneliness [54], suggesting that patients who were less well adjusted to the role changes caused by cancer had higher loneliness scores.

In conclusion, the health status that affected patients with social isolation could be categorized into three aspects: physical health status, psychological health status, and social health status. All of these aspects exhibited significant correlations with social isolation. For instance, a deteriorating physical condition including some detailed symptoms may predict a higher degree of social isolation. Moreover, patients with other poor health statuses such as impaired mental state and role function may be more vulnerable to experiencing heightened levels of social isolation.

#### 3.4.4. Coping

Coping styles, as one of the influencing factors of social isolation, will be thoroughly discussed. Only one quantitative study collected data on the coping styles of cancer patients. In a cross-sectional study on breast cancer patients, Wang and colleagues suggested that coping style was one of the influencing factors of social isolation. As the findings of their study showed, avoidance and yield coping could positively predict social isolation in breast cancer survivors, while confronting coping could negatively predict loneliness [67]. The results from a qualitative study on patients with breast cancer demonstrated that withholding truth or projecting images that they perceived as inauthentic would contribute to their loneliness [50].

In short, negative coping was a risk factor for social isolation, while positive coping was a protective factor.

#### 3.4.5. Social Support and Social Interaction

A total of 18 of the included studies revolved around the social aspects of functioning. There was an inverse correlation between subjective or/and objective social isolation and (perceived) social support or emotional support [10,22,48,49,51,53,54,61,62,63,64,65], and a positive relationship between loneliness and social constrain [52,59]. In addition, social interaction was proven to be associated with subjective social isolation [49,51,56,58,66,73], confirming that lower levels of social network diversity or higher scores of objective social isolation were associated with elevated loneliness.

In general, social support and social interaction were positively related to a lower degree of social isolation, while social constraint, as negative social interaction [74], was positively correlated with the level of loneliness.

## 4. Discussion

### 4.1. Influencing Factors of Social Isolation

#### 4.1.1. Demographic Characteristics

The effects of age and gender on social isolation were at variance. Regarding age, younger patients experience heightened stress related to work and personal life [20], which would lead to social isolation. However, older patients were more prone to objective social isolation and had limited social support [75], which may add up to feelings of isolation over time. As for gender differences, it is plausible that compared to male patients, female patients display a greater willingness to express their emotion and have more outlets to release negative emotion [55]. But, at the same time, females tend to be more emotional. These differences deserve more exploration in the future. 

The possible explanation for negative relationships between social isolation and educational level [53,56,65,67,68] is that patients with higher education levels are also more likely to accept health education and medical guidance, as well as be better able to discern exaggerated information on the Internet in order to reduce misunderstanding. Furthermore, patients with higher education levels tend to have better psychological adjustment, enabling them to cope more effectively with the stress and anxiety accompanying chronic illnesses [76].

The Type D personality is characterized by a tendency towards depression [77]. Individuals with this personality trait often exhibit heightened sensitivity to the stressors associated with cancer, leading to increased negative emotions. Furthermore, concerns about potential discrimination and prejudice due to changes in physical appearance resulting from treatment may lead patients to withdraw from their social support networks [78]. Additionally, individuals with a Type D personality may experience discomfort and apprehension in social interactions, maintaining emotional distance from family members, medical professionals, and fellow patients. This social inhibition would hinder individuals access to necessary support during their periods of depression, resulting in internalized emotions and increased social isolation. 

One study indicated that a patient’s occupational status influenced their isolation scores, suggesting that unemployed patients had higher social isolation scores [67]. Employed patients would have more opportunities for interaction with others and derive a sense of accomplishment from their work. Additionally, cancer places a significant economic burden on public health systems [79]. Despite the presence of medical insurance, cancer patients still incur personal costs [80]. Employed patients have greater financial capacity to handle treatment-related burdens such as expenses. Similarly, patients with higher household incomes would experience less financial burden and not expend too much energy reducing their economic burden. Additionally, a possible explanation for the negative association between social isolation and family income [10,63,73] is that patients with higher levels of education and family incomes are more likely to have a strong support system, including emotional support from friends and family. This support can significantly alleviate the stress and isolation often associated with chronic illnesses, leading to a more positive outlook on life and improved overall well-being.

Coping with cancer is a difficult process, and the process of coping with cancer is long and winding. Therefore, accompanying itself would be powerful. The absence of children or living alone may also contribute to the increased likelihood of social isolation among cancer patients compared to those with children or who do not live alone. In addition, the results all confirmed that married patients had lower scores of loneliness [53,57,59,66,67,71,73], which aligns with the findings reported in a 2014 review [14]. In addition to providing vital companionship, a spouse, being a crucial source of social support [81], plays an indispensable role in helping cancer patients with coping with the disease more effectively. Furthermore, spouses often assume caregiving responsibilities and provide life care, spiritual comprehension, and companionship to ensure that cancer patients perceive the necessary support, which may significantly alleviate the burden experienced by cancer patients. However, spouses should pay careful attention to their supportive and protective behaviors towards the patients. According to Bodenmann’s systemic transactional model [82], dyadic coping refers to the common reactions and strategies of both spouses in the face of stressful events. It is important to note that overprotection as a form of negative coping is not conducive to the recovery of patients, resulting in role strengthening, over-dependence on caregivers, and an increased burden of care. 

Professionals should pay attention to cancer patients with the aforementioned demographic factors. These patients may be particularly vulnerable and experience more feelings of isolation due to their unique circumstances. Therefore, professionals should not only provide necessary medical care but also offer enhanced emotional support and encouragement to address the specific needs of these patients.

#### 4.1.2. Having Cancer

According to our review, type and stage of disease and treatment could affect the feeling of isolation. However, there is currently a lack of scholarly consensus on this particular aspect. For example, there were significantly different scores of loneliness in a research of urological cancer patients [71]. The possible explanation is that some bladder cancer patients even need to carry urine pouches throughout their lives, while prostate and renal cancer patients do not need to carry urine pouches. The existence of a urine pouch would increase patients’ feelings of isolation. Given the varying types of treatment for different cancers, further exploration of the stage of disease and type and stage of treatment specific to each cancer is necessary. 

Findings about treatment-related time were controversial. One possible explanation for the positive association [55] is that as treatment progresses, the level of social concern and support for cancer patients tends to decrease. This decline in support could potentially result in an increased sense of social isolation for those who receive less assistance, as they may feel abandoned or neglected [83]. The opposite findings [56,63,65,67] may be attributed to the gradual process of adjustment and acceptance that cancer patients undergo as they come to terms with their illness. As patients become more comfortable with their condition and their functional abilities improve, they may be more willing to reintegrate into society and attempt to resume their normal lives. Although the association requires further exploration to determine the changing picture of social isolation at various stages, there is no doubt that patients require support and assistance at all stages of their disease to adapt to role changes, which would impact the score of social isolation [54]. 

Additionally, the time since diagnosis, as another important duration, also needs to be analyzed. Similar to the above analysis, the patient’s own coping style or ability to regulate emotions, as well as their important social support system, can change with the time since diagnosis. However, in six studies, no relationship was found between the time since diagnosis and social isolation [59,60,61,62,64,72].

Our findings indicated that the informed status of a disease had an impact on the level of social isolation [54,68], which may be attributed to the scare of disease progression and death. However, attitudes toward informing patients about the condition of family members vary from one another [84,85]. Professionals should carefully consider this aspect in their clinical practice, including when considering when and how to inform patients about their condition, in order to minimize negative emotions such as loneliness. What is more, the informed status would be divided into kinds of statuses, including partially informed status and fully informed status. Patients who are fully aware of the disease often appear more confident and cooperative with treatment, while patients who are not clear about the disease often show great concern about its treatment and the trend of disease progression. The relationship between specific levels of informed status and social isolation among cancer patients deserves further exploration.

All in all, under the stressful circumstance of cancer, cancer itself and its related treatment take a toll on patients. According to the conceptual framework of group intervention [34], the provision of social support from various sources is associated with a reduction in cancer-related stress. Therefore, it is crucial for healthcare professionals to provide necessary and needed disease-related information to cancer patients, aid patients in comprehending their condition properly, even address the topic of mortality to alleviate their scare of death in their clinical practice, encourage open communication with peers and participate group activities as long as physical conditions permit, as well as encouraging the family members to offer continuous support.

#### 4.1.3. Health Status

Poor physical status was proven to positively correlate with the level of isolation [51,56,59]. One plausible explanation is that individuals with poor physical status often lack the necessary vitality to engage in group activities, leading them to opt to stay at home. Consequently, they have limited opportunities for social interaction. This objective social isolation, in turn, exacerbates feelings of loneliness. 

The findings regarding the correlation between social isolation and psychological status, both negative and positive, were consistent and unambiguous. The results demonstrated a positive correlation between negative mental status [22,48,51,62,65,66,69,70], typified by anxiety and depression, and the degree of social isolation. This correlation could be attributed to the fact that individuals with negative mental health tend to engage in fewer social activities [22], leading to a decreased level of social integration. Consequently, they often experience a heightened sense of isolation. According to the framework presented [34], there existed a reciprocal relationship between health status and coping mechanisms, whereby patients with positive psychological status were found to be more inclined to break through difficulties rather than avoid them. A positive mental situation is beneficial for social connection, and it fosters the willingness to communicate and engage with others [86]. This proactivity in social interaction, in turn, helps to alleviate feelings of isolation and loneliness [87]. So, family members and professionals should pay attention to the patient’s mental status and assist the patient in avoiding their passive emotions. Family members, such as the primary caregiver, should be aware of the impact of the mental status. They should listen to the patients carefully, communicate proactively, and share interesting things with them. The care of a family member with cancer often involves the provision of various forms of support, including emotional and physical assistance [88]. Given the complexity and time commitment required for this caregiving role, family caregivers may struggle to adapt, leading to high levels of loneliness as they feel disconnected from their usual support network [89], which would reduce the quality of the support provided. Therefore, it is vital for caregivers themselves to maintain a good mental state; thereafter, they can create a positive and relaxed family atmosphere, which is beneficial for cancer patients to feel warmth and love psychologically, as well as to fight against the disease in a good psychological state. Professionals, such as doctors and nurses, should identify any signs of negative emotions such as anxiety and depression, and perform early active psychological intervention. 

Regarding role function as an indicator of social well-being, our findings suggested a negative correlation between role function and social isolation [54]. When cancer patients become ill, they might struggle to adapt to their new patient roles while finding it challenging to promptly and correctly adjust to various previously assumed societal roles. Consequently, these patients may develop more negative emotions and experience increased levels of social isolation.

According to previous reviews and our study, health status and social isolation interact with each other [90]. Social isolation causes adverse health status, which, in turn, engenders increased social isolation.

From this perspective, healthcare professionals should pay attention to a patient’s primary complaints by striving to alleviate adverse symptoms effectively while assisting them in adapting to their patient roles at the earliest opportunity in order to minimize adverse emotional experiences. In the midst of their noble calling, these medical personnel attending to the patient are not immune to the emotional and mental toll that comes with the job. Each one of them is an individual with their own unique emotions, vulnerabilities, and personal struggles [91]. While they may feel deep frustration and empathy towards the patient’s suffering, it is crucial for them to effectively manage these emotions in order to avoid burnout and ensure the provision of high-quality care [92]. Otherwise, they risk being consumed by the weight of their duties, leading to emotional exhaustion and potentially affecting their ability to provide the best possible care for their patients [93].

#### 4.1.4. Coping

According to stress coping theory [38], when individuals perceive stressors, they respond after primary and secondary assessments. What is more, individuals exhibit a range of psychological and behavioral responses when faced with stress [38]. Coping can be categorized into positive coping and negative coping. Negative coping adversely affects individual well-being [94], while positive coping mitigates the detrimental impact of various stressful events on patients’ psychology and cognition, thereby enhancing their quality of life [95]. Patients who engage in negative coping such as withdrawal experience diminished interest and energy in their surroundings, leading to decreased social integration. Conversely, employing positive coping strategies like problem-solving and self-consolation enables individuals to better adapt to stress and minimizes its impact on psychological and cognitive functioning, thus improving social skills and interpersonal relationships. Our review indicates a correlation between coping and social isolation [50,67]. Therefore, it is recommended that patients cultivate a positive mindset and adopt positive coping strategies to reduce the adverse effects of stress on individual psychological and physical health and promote the good performance of individuals in social interactions. In addition, professionals should pay attention to the coping styles of patients in order to provide them with targeted interventions and support to help them better cope with the stresses and challenges in life.

Given that spousal caregivers would experience the cancer diagnosis and cancer treatment of their loved one as a great strain, dyadic coping would occupy an important position in fighting cancer. Dyadic coping emphasizes joint decision-making and interactions between both parties in the face of stressful events. It is seen as a systematic way of coping that focuses not only on individual responses but also on interactions between the two parties [82]. Compared with individual coping, dyadic coping would help to researchers take a more comprehensive view of outcomes for cancer patients and their spousal caregivers. This issue of the association between dyadic coping and social isolation among cancer patient–spousal caregiver dyads is an intriguing one that could be usefully explored in future research.

#### 4.1.5. Social Support and Social Interaction

As in the 2014 review [14], the results in this review demonstrated that patients who accepted more social support and engaged in more social interaction experienced lower levels of social isolation. 

According to the definition of loneliness as a subjective component of social isolation, social loneliness can be effectively mitigated through the provision of structural social support, which primarily aims to enhance the quantity of social interactions. This is because, in the context of structural social support, individuals are often provided more opportunities to engage in activities and communicate with others. Additionally, objective social isolation would also be alleviated by increasing the quantity of social interaction. On the other hand, emotional loneliness may decrease with functional social support, which focuses on improving the quality of social relationships. In this way, it would be constructive to offer adequate social support to cancer patients experiencing social isolation or loneliness.

Social support plays a crucial role in coping with the emotional challenges associated with cancer. Having someone to talk to and share their fears and concerns with can provide immense comfort and reassurance [96]. It allows patients to express their thoughts openly without judgment or criticism, which can be incredibly therapeutic. Additionally, research suggests that strong social connections can positively influence treatment outcomes for patients [97]. The availability of social support can help patients to establish and maintain social ties, thereby reducing psychological distress among cancer patients by providing an outlet for emotional expression and offering guidance on stress management techniques [98]. This ultimately contributes to improving mental health outcomes and quality of life.

Various sources contribute to social support, including peers, family members, and professionals. In addition to its positive impact on quality of life and overall well-being, perceived social support has been found to have numerous benefits for cancer patients [99]. Social support from peers provides a sense of belonging and connection with others who are going through similar experiences, and the encouragement and motivation received from loved ones can give patients the strength to overcome illness [100], which can help to alleviate feelings of isolation that often accompany a cancer diagnosis and reduces the risk of social isolation [96,100]. Informal and formal social networks both play a crucial role in providing support, thus avoiding experiences of helplessness and isolation [101]. However, perceived social support would be influenced by various factors such as mental status, with individuals possessing an optimistic mindset potentially perceiving greater levels of support than what is actually being offered [102]. Given that cancer represents a highly distressing event impacting both physical and psychological health, it is worthwhile to investigate whether the compromised health statuses of patients contribute to a decline in perceived social support and potentially promote social isolation.

According to our review and analysis based on the framework of group intervention [34], social support plays a crucial role in mitigating the impact of cancer diagnosis. This includes tangible assistance, as well as intangible forms such as valuable information and encouragement from family members, peers, and healthcare professionals, which also promotes health status and improves coping. 

For clarity, the above-integrated discussion is presented in Table 3.

### 4.2. Limitation

There is still a need to acknowledge some limitations existing in this review. Firstly, there are language biases as studies conducted in languages other than English or Chinese were not included. Secondly, the measurement tools employed to assess social isolation and loneliness lack complete consistency across different studies, potentially impacting the accuracy of our assessment. Thirdly, current studies on social isolation have not mainly focused on both objective and subjective aspects. However, social isolation is a multi-dimensional concept, which includes objective social isolation and perceived social isolation. Lastly, due to a multitude of internal adjustment factors utilized in various studies and inconsistencies among these factors across studies, the source of heterogeneity is challenging to identify. Furthermore, potential unknown confounders may still be present within our present analysis.

### 4.3. Recommendations for Future Research

This study meticulously analyzed a broad range of studies, examining the intricate factors that were associated with social isolation among cancer patients. It is widely known that caregivers play an indispensable role in the cancer treatment process, and they have a critical influence on the patient’s mental and emotional well-being by providing various types of support. However, the existing literature fails to provide a comprehensive understanding of the relationship between social isolation among cancer patients and caregiver-related variables due to the limited scope of the studies available, resulting in inconclusive findings and fragmented insights. To enrich the existing body of knowledge, an increased focus on patient–caregiver dyads in future research is recommended. By examining the interactions and dynamics between cancer patients and their caregivers, a deeper understanding of the factors influencing social isolation and the beneficial interventions that can mitigate its negative effects can be gained. 

Our review of influencing factors is based on Stewart’s framework [34], which focused on variables that affect individuals’ reactions to support group intervention. This framework has served as a valuable guide in understanding influencing factors of social isolation and improving social isolation. 

Based on this framework, we propose designing a group psychological intervention specifically tailored for cancer patients experiencing social isolation. This innovative intervention program aims to address the multifaceted needs of these patients by providing them with both peer and professional support. Through this psychological intervention program, cancer patients will not only receive valuable emotional support from their peers who share similar experiences but also benefit from professionals who can provide valuable guidance and resources. This dual-layer approach to social support is expected to have a profound impact on patients’ overall well-being. Moreover, this psychological group intervention will address various aspects of patients’ lives, including their health statuses, coping strategies, and social support and interaction, thereby reducing social isolation.

### 4.4. Clinical Implication

The diagnosis and treatment of cancer places significant emotional demands on patients and their families, impacting not only their coping abilities but also their broader support networks such as friends. Complications during treatment can lead to adverse psychological reactions like anxiety and depression, resulting in decreased physical, psychological, and social adaptability for patients. Social isolation is a common form of social maladjustment experienced by cancer patients [4,103]. Previous reviews have shown that cancer patients experience moderate levels of loneliness [14]. Therefore, addressing social maladjustment such as social isolation related to cancer influences is crucial.

This review analyzed and synthesized the influencing factors of social isolation among cancer patients. It is imperative for healthcare professionals to identify factors that may be contributing to patients’ social isolation, recognize the varying degrees of social isolation experienced by these patients, and develop targeted interventions to alleviate their plight. By raising awareness about the issue and encouraging their family to engage in the companion, we can help cancer patients to feel less isolated and improve their overall well-being.

## 5. Conclusions

In this systematic review, we aimed to identify the influencing factors of social isolation in cancer patients and classify them into demographic characteristics, having cancer, health status, coping, and social support and social interaction. However, these factors have not been agreed upon. So, we proposed that longitudinal studies with ample samples in the future are needed. Meanwhile, the group positive psychological intervention should be taken into account as a means to alleviate the level of social isolation.

## Figures and Tables

**Table 1:** Quality assessment table according to MMAT.

Qualitative	1.1. Is the Qualitative Approach Appropriate to Answer the Research Question?	1.2. Are the Qualitative Data Collection Methods Adequate to Address the Research Question?	1.3. Are the Findings Adequately Derived from the Data?	1.4. Is the Interpretation of Results Sufficiently Substantiated by Data?	1.5. Is There Coherence between Qualitative Data Sources, Collection, Analysis, and Interpretation?
Dong et al., 2022 [48]	yes	yes	yes	yes	yes
Ettridge et al., 2018 [49]	yes	yes	yes	yes	yes
Rosedale et al., 2009 [50]	yes	yes	yes	yes	yes
** Quantitative descriptive **	** 4.1. Is the sampling strategy relevant to address the research question? **	** 4.2. Is the sample representative of the target population? **	** 4.3. Are the measurements appropriate? **	** 4.4. Is the risk of nonresponse bias low? **	** 4.5. Is the statistical analysis appropriate to answer the research question? **
Adams et al., 2017 [51]	yes	yes	yes	yes	yes
Adams et al., 2017 [52]	yes	yes	yes	yes	yes
Çamlıca and Koç, 2022 [53]	yes	yes	yes	yes	yes
Chen et al., 2022 [54]	yes	yes	yes	yes	yes
Choi and Henneghan, 2022 [55]	yes	yes	yes	cannot tell	yes
DuanMu et al., 2022 [56]	yes	yes	yes	cannot tell	yes
Hao et al., 2023 [57]	yes	yes	yes	cannot tell	yes
He et al., 2023 [10]	yes	yes	yes	yes	yes
Hill & Frost. 2022 [58]	yes	yes	yes	cannot tell	yes
Hyland et al., 2019 [59]	yes	yes	yes	yes	yes
Kavalalı Erdoğan and Koç, 2021 [60]	yes	yes	yes	yes	yes
Kömürcü et al., 2014 [61]	yes	yes	yes	cannot tell	yes
Kosugi et al., 2021 [62]	yes	yes	yes	yes	yes
Liang et al., 2022 [63]	yes	yes	yes	yes	yes
Lin et al., 2023 [64]	yes	yes	yes	yes	yes
Liu et al., 2021 [22]	yes	yes	yes	yes	yes
Liu et al., 2021 [65]	yes	yes	yes	cannot tell	yes
Miaskowski et al., 2021 [66]	yes	yes	yes	no	yes
Wang et al., 2020 [67]	yes	yes	yes	yes	yes
Wang et al., 2021 [68]	yes	yes	yes	cannot tell	yes
Wang et al., 2022 [69]	yes	yes	yes	cannot tell	yes
White et al., 2023 [70]	yes	yes	yes	yes	yes
Xia et al., 2023 [71]	cannot tell	yes	yes	cannot tell	yes
Zhang et al., 2022 [72]	yes	yes	yes	cannot tell	yes
** Combined Quantitative and Qualitative Studies **	** 5.1. Is there an adequate rationale for using a mixed methods design to address the research question? **	** 5.2. Are the different components of the study effectively integrated to answer the research question? **	** 5.3. Are the outputs of the integration of qualitative and quantitative components adequately interpreted? **	** 5.4. Are divergences and inconsistencies between quantitative and qualitative results adequately addressed? **	** 5.5. Do the different components of the study adhere to the quality criteria of each tradition of the methods involved? **
Clifton et.al, 2022 [73]	yes	yes	yes	yes	yes

**Table 2:** Summary of studies on cancer patients: influencing factors of social isolation among cancer patients.

Qualitative Studies				
Authors, Year, Country [Citation Number]	Aims	Informants	Instrument Used	Key Findings and Classifications of Influencing Factors
Dong, 2022, China [48]	To describe social isolation-related psychological experiences of cancer patients in order to provide practicable references for developing relevant nursing support programs.	20 cancer patients	Semi-structured interview	Health status: physical health status: cancer patients suffered social isolation because of their impaired physical health; psychological health status: cancer patients suffered social isolation because of their poor psychological status. Social support and social interaction: cancer patients suffered social isolation because of their insufficient social support.
Ettridge, 2018, Australia [49]	To provide in-depth insight into men’s experiences of prostate cancer, specifically perceived stigma and self-blame, social isolation, unmet needs, and help-seeking behavior.	20 men diagnosed with prostate cancer	Semi-structured interview	Health status: physical health status: many participants experienced feelings of loneliness due to physical consequences of treatment and side effects. Social support and social interaction: many participants experienced feelings of loneliness due to a lack of readily available support/social contact, reluctance to talk to others, and perceived withdrawal from others.
Rosedale et al., 2009, USA [50]	To describe the experience of loneliness for women more than a year following breast cancer treatment.	13 women, 1–18 years following breast cancer treatment.	Open-ended interviews	Health status: psychological health status: feeling alone in the awareness of mortality; coping: wi thholding truth or projecting images that they perceived as inauthentic contributed to the loneliness.
**Quantitative Studies**				
**Authors, Year, Country** **[Citation Number]**	**Aims**	**Samples**	**Instrument Used**	**Key Findings and Classifications of Influencing Factors**
Adams et al., 2017, USA [51]	To develop and validate the cancer loneliness scale and cancer-related negative social expectation scale.	186 cancer patients	The 20-item UCLA Loneliness Scale-Version 3 (UCLA-V3); the emotional support, depressive and anxiety symptoms, and physical quality of life subscales (4 items each) from PROMIS measures; The mental health and physical health subscales (4 items each) from the 10-item Global Health measure; a 3-item scale adapted from the Social Network Index	Health status: physical health status: physical quality of life was negatively correlated with loneliness (*p* < 0.01); psychological health status: anxiety and depression were positively correlated with loneliness (*p* < 0.01), and mental quality of life was negatively correlated with loneliness (*p* < 0.01). Social support and social interaction: emotional support and the number of relatives and friends with whom participants had regular contact were negatively correlated with loneliness (*p* < 0.01).
Adams et al., 2018, USA [52]	To examine whether cancer-related loneliness mediated the relationships between social constraints and symptoms in patients with various cancers.	182 cancer patients	7-item Cancer Loneliness Scale; 5-item version of the Social Constraints Scale; Patient-Reported Outcomes Measurement Information System (PROMIS) measures	Social support and social interaction: social constraint on cancer-related disclosure was positively correlated with loneliness *(p* < 0.001).
Çamlıca and Koç, 2022, Turkey [53]	To determine the relationships between the perceived loneliness and social support levels of Turkish oncology patients, as well as their quality of life and symptom management.	370 cancer patients	10-item UCLA loneliness scale (version 3); 12-item Multi-Dimensional Scale of Perceived Social Support (MSPSS); 27-item FACT-G Quality of Life Scale (Version 4); The Edmonton Symptom Assessment Scale	Demographic characteristics: gender: male patients had higher loneliness; marital status: married patients had lower loneliness; education level was negatively correlated with loneliness (all *p* < 0.05). Health status: physical health status: A positive significant relationship between loneliness and fatigue (r = 0.296, *p* < 0.01), pain (r = 0.193, *p* < 0.01), sleeplessness (r = 0.199, *p* < 0.01), nausea (r = 0.243, *p* < 0.01), loss of appetite (r = 0.244, *p* < 0.01), and shortness of breath (r = 0.220, *p* < 0.01) was found; psychological health status: a positive significant relationship between loneliness and sadness (r = 0.246, *p* < 0.05), worry (r = 0.250, *p* < 0.01), and feeling unwell (r = 0.376, *p* < 0.01) was found. Social support and social interaction: social support was negatively correlated with loneliness (r = −0.754, *p* < 0.01).
Chen et al., 2022, China [54]	To investigate the status quo and influencing factors of loneliness in hospitalized cancer patients.	313 hospitalized cancer patients	20-item UCLA Loneliness Scale-Version 3 (UCLA-V3); 12-item Perceived Social Support Scale (PSSS); 30-item European Organization for Research and Treatment of Cancer Core Quality of Life Questionnaire (EORTC QLQ-C30)	Demographic characteristics: gender: female patients had higher loneliness (β = 1.74, *p* < 0.001). Having cancer: the informed situation was positively correlated with loneliness (β = 2.20, *p* < 0.001). Health status: physical health status: nausea and vomiting were positively correlated with loneliness (β = 0.03, *p* < 0.001); psychological health status: emotional function was negatively correlated with loneliness (β = −0.10, *p* < 0.001); social health status: role function was negatively correlated with loneliness (β = −0.06, *p* < 0.001). Social support and social interaction: social support was negatively correlated with loneliness (β = −0.27, *p* < 0.001).
Choi and Henneghan, 2022, USA [55]	To compare the severity of psychosocial outcomes (loneliness, perceived stress, depressive symptoms, anxiety, fatigue, and daytime sleepiness) between younger (aged less than 50 years) and older (aged 50 years or older) BCS who completed chemotherapy 6 months to 10 years prior and identify predictors of loneliness for younger BCS.	90 breast cancer patients	20-item UCLA Loneliness Scale-Version 3 (UCLA-V3); 8-item Patient-Reported Outcomes Measurement Information System Emotional Distress–Anxiety, –Depression, and–Fatigue Short Forms; 8-item Epworth Sleepiness Scale; 8-item Epworth Sleepiness Scale; 10-item Perceived Stress Scale	Demographic characteristics: age was negatively correlated with loneliness (*p* < 0.001); not having children was positively correlated with loneliness (β = −0.443, *p* = 0.001). Having cancer: a longer time since the completion of chemotherapy was positively correlated with loneliness (β = 0.328, *p* = 0.012).
Duan Mu et al., 2022, China [56]	To investigate the current status of social isolation and its influencing factors in 242 elderly patients with colostomy for colorectal cancer in Zhengzhou city.	242 elderly patients with colostomy of colorectal cancer	15-item General Alienation Scale (GAS); 25-item impact on participation and autonomy questionnaire (IPA); 17-item Social Relationship Quality Scale (SRQS); 10-item modified Barthel Index (MBI)	Demographic characteristics: gender: female patients had higher feelings of isolation (β = 2.631, *p* = 0.040); education level was negatively correlated with subjective social isolation (β = −3.284, *p* = 0.003). Having cancer: postoperative time (β = −3.726, *p* = 0.005) and daily living ability (β = −0.280, *p* < 0.001) were negatively correlated with subjective social isolation. Social support and social interaction: social participation (β = 2.804, *p* < 0.001) and social relationship quality (β = −0.682, *p* < 0.001) were negatively correlated with subjective social isolation.
Hao et al., 2023, China [57]	To understand the status quo of social isolation in ovarian cancer patients accepting postoperative chemotherapy and analyze its influencing factors.	194 ovarian cancer patients accepting postoperative chemotherapy	14-item Type D Personality Scale-14 (DS14); 12-item Perceived Social Support Scale (PSSS); 15-item General Alienation Scale (GAS)	Demographic characteristics: personality: type D personality was a risk factor for subjective social isolation (β = 0.185, *p* = 0.005); marital status: married patients experienced lower subjective social isolation (β = 0.358, *p* < 0.001); age was positively correlated with subjective social isolation (β = 0.305, *p* < 0.001). Having cancer: disease stage: patients with late-stage disease had high levels of subjective social isolation (β = 0.166, *p* = 0.003).
He et al., 2023, China [10]	To investigate the social isolation subtypes of patients with breast cancer (BC) and explore its influencing factors.	303 women with breast cancer	20-item Chinese version of the Loneliness Scale (C-LS); 6-item Chinese version of the Social Anxiety Scale (C-SAS); 14-item Chinese version of the Social Avoidance and Distress Scale (C-SADS); 5-item Family APGER Index; 15-item Chinese version of the Self-Transcendence Scale (C-STS)	Demographic characteristics: monthly family income was negatively correlated with social isolation (*p* < 0.001). Having cancer: patients accepting surgery or chemotherapy were more likely to suffer from social isolation (*p* < 0.001). Health status: psychological health status: levels of self-transcendence were negatively correlated with social isolation (*p* < 0.001). Social support and social interaction: family function was negatively correlated with social isolation (*p* < 0.001).
Hill and Frost. 2022, USA [58]	To examine variables that might be associated with elevated loneliness and play a role in the loneliness–psychological distress relationship among women with ovarian cancer.	125 women with ovarian cancer	20-item UCLA Loneliness Scale-Version 3 (UCLA-V3); the depressive and anxiety symptoms subscales (7 items each) from the Depression Anxiety Stress Scales (DASS-21); 10-item The Self-Perceived Burden Scale (SPBS); The Social Network Index (SNI); Two subscales of the COPE	Health status: psychological health status: self-perceived burden was positively correlated with loneliness (*p* < 0.001). Social support and social interaction: the level of social network diversity was negatively correlated with loneliness (*p* < 0.001).
Hyland et al., 2019, USA [59]	To investigate the relationship between loneliness, depressive symptoms, quality of life, and social cognitive variables (e.g., stigma, social constraint, cancer-related negative social expectations) and explore loneliness as a mediator of the relationship between social cognitive variables and depressive symptoms and quality of life in lung cancer patients beginning treatment.	105 lung cancer patients	20-item UCLA Loneliness Scale-Version 3 (UCLA-V3); 21-item Cataldo Lung Cancer Stigma Scale-Shortened Version (CLCSS-SV); 15-item Social Constraint Scale-Cancer Version (SCS-CV); 5-item Cancer-related Negative Social Expectation Scale (CNSES); 37-item Functional Assessment of Cancer Therapy-Lung (FACT-L); 20-item Center for Epidemiological Studies Depression Scale (CES-D)	Demographic characteristics: marital status: being unmarried was associated with greater loneliness (*p* < 0.05); smoking status: current smokers reported greater loneliness than non-current smokers (*p* < 0.05). Health status: physical health status: performance status was negatively correlated with loneliness (*p* < 0.05); psychological health status: stigma and negative social expectation were positively correlated with loneliness (all *p* < 0.001). Social support and social interaction: social constraint was positively correlated with loneliness (*p* < 0.001).
Kavalalı Erdoğan and Koç, 2021, Turkey [60]	To determine the relationships among loneliness, death perception, and spiritual well-being in adult oncology patients.	347 cancer patients	10-item UCLA Loneliness Scale(version 3); 15-item Personal Meanings of Death Scale; 12-item Functional Assessment of Chronic Illness Therapy–Spiritual Well-being Scale (FACIT-Sp)	Health status: psychological health status: spiritual well-being was negatively correlated with loneliness (r = −0.217, *p* < 0.01).
Kömürcü et al., 2014, Turkey [61]	To determine the impact of illness on marriage and the level of loneliness for women diagnosed with gynecologic cancer.	95 women with gynecologic cancer	20-item UCLA loneliness scale	Demographic characteristics: age was negatively correlated with loneliness (*p* = 0.006). Having cancer: cycle of chemotherapy was positively correlated with loneliness (*p* = 0.049). Social support and social interaction: patients who perceived isolation from their spouse had higher loneliness than the other patients (*p* = 0.007).
Kosugi et al., 2021, Japan [62]	To investigate the association between loneliness and the frequency of using online peer support groups among cancer patients with minor children.	334 cancer patients with minor children	20-item UCLA loneliness scale (version 3); 6-item Lubben Social Network Scale (LSNS-6); 6-item K6 scale	Health status: psychological health status: distress was positively correlated with loneliness (OR = 1.16, 95% *CI* 0.73–0.83). Social support and social interaction: so cial networks and frequent use of online peer support groups were negatively correlated with loneliness (OR = 0.78, 95% *CI* 1.09–1.23).
Liang et al., 2022, China [63]	To investigate the status and influencing factors of social isolation in cervical cancer survivors and provide a reference for implementing targeted intervention measures.	363 cervical cancer patients	15-item General Alienation Scale (GAS); 24-item Social Impact Scale (SIS); 10-item Social Support Rating Scale (SSRS)	Demographic characteristics: monthly family income was negatively correlated with subjective social isolation (β = −2.371, *p <* 0.001). Having cancer: time since last treatment was negatively correlated with subjective social isolation (β = −2.538, *p <* 0.001). Health status: psychological health status: stigma was positively correlated with subjective social isolation (β = 0.120, *p <* 0.001). Social support and social interaction: soc ial support was negatively correlated with subjective social isolation (β = −0.284, *p <* 0.001).
Lin et al., 2024, China [64]	To identify the factors associated with loneliness among patients with cancer in China.	205 cancer patients	7-item Cancer Loneliness Scale (CLS); 10-item Social Support Rating Scale (SSRS); 14-item Hospital Anxiety and Depression Scale (HADS); 5-item Cancer-Related Negative Social Expectations Scale (C-rNSES)	Health status: psychological health status: depression (β = 0.262, *p* = 0.001) and negative social expectation (β = 0.327, *p <* 0.001) were positively correlated with loneliness. Social support and social interaction: social support was negatively correlated with loneliness (β = −0.294, *p <* 0.001).
Liu et al., 2021, China [22]	To explore the correlations among social isolation and symptoms of anxiety and depression among patients with breast cancer in China and further verify the mediating role of social support in social isolation and symptoms of depression or anxiety.	389 female inpatients diagnosed with breast cancer	14-item Hospital Anxiety and Depression Scale; 10-item Social Support Rating Scale; 6-item social isolation scale with reference from the simplified version of Lubben’s Social Network	Health status: psychological health status: anxiety (r = 0.369, *p <* 0.01) and depression (r = 0.466, *p <* 0.01) were positively correlated with objective social isolation. Social support and social interaction: social support was negatively correlated with objective social isolation (r = −0.223, *p <* 0.01).
Liu et al., 2021, China [65]	To investigate the status of social isolation among lung cancer survivors and analyze its influencing factors.	228 lung cancer survivors	15-item General Alienation Scale (GAS); 10-item Self-Esteem Scale (SES); 12-item Perceived Social Support Scale (PSSS)	Demographic characteristics: education level was negatively correlated with subjective social isolation (β = −2.296, *p <* 0.001). Having cancer: time after cure was negatively correlated with subjective social isolation (β = −3.204, *p <* 0.001). Health status: psychological health status: self-esteem was negatively correlated with subjective social isolation (β = −0.432, *p <* 0.001). Social support and social interaction: perceived social support was negatively correlated with subjective social isolation (β = −0.217, *p <* 0.001).
Miaskowski et al., 2021, USA [66]	To determine the prevalence of loneliness in a sample of oncology patients; evaluate differences in demographic, clinical, and symptom characteristics between lonely and nonlonely patients; and determine which demographic, clinical, and symptom characteristics were associated with membership of the lonely group.	606 cancer patients	20-item UCLA Loneliness Scale-Version 3 (UCLA-V3); 6-item Social Isolation Scale (SIS); Center for Epidemiological Studies–Depression scale (CES- D); Spielberger State-Trait Anxiety Inventories (STAI-S, STAI-T); General Sleep Disturbance Scale (GSDS); Lee Fatigue Scale (LFS); Attentional Function Index; Brief Pain Inventory	Demographic characteristics: marital status: married patients had lower loneliness (OR = 2.94, 95% *CI* 1.69–5.00). Health status: psychological health status: anxiety (OR = 3.17, 95% *CI* 1.86–5.39) and depression (OR = 3.24, 95% *CI* 1.85–5.67) were positively correlated with loneliness. Social support and social interaction: obje ctive social isolation was positively correlated with loneliness (OR = 0.66, 95% *CI* 0.60–0.72).
Wang et al., 2020, China [67]	To study the status quo and influencing factors of social isolation among breast cancer survivors and provide a reference for future nursing interventions for this group.	228 breast cancer patients	20-item UCLA Loneliness Scale; 28-item Social Avoidance Scale (GAS); 6-item Social Anxiety Scale (SAS); 20-item modified Medical Coping Modes Questionnaire (MCMQ)	Demographic characteristics: age and education level were negatively correlated with social isolation; marital status: married patients experienced lower social isolation; occupation status: patients who were unemployed or on sick leave had higher scores for social isolation compared to working and retired cancer survivors (all *p* < 0.05). Having cancer: operation mode: patients who underwent radical mastectomy had higher social isolation scores than those who underwent breast-conserving therapy; postoperative time was negatively correlated with social isolation (all *p* < 0.05). Coping: avoidance and yield coping were positively correlated with social isolation (all *p* < 0.05).
Wang et al., 2021, China [68]	To investigate the clinical characteristics of loneliness in patients from the oncology department.	344 cancer patients	20-item UCLA Loneliness Scale-Version 3 (UCLA-V3)	Demographic characteristics: gen der: female patients had higher loneliness (*p* < 0.001); residence: rural patients had higher loneliness than country and urban patients (*p* = 0.005); education level was negatively correlated with loneliness (*p* = 0.006); medical insurance: patients who bought new rural medical insurance felt more loneliness than those who bought resident medical insurance, employee medical insurance, and business insurance (*p* = 0.001). Having cancer: treatment stage: patients accepting chemotherapy had higher loneliness (*p* < 0.001); awareness of diagnosis: informed patients had more loneliness than ignorant patients (*p* = 0.001).
Wang et al., 2022, China [69]	To study the status and influencing factors of social isolation among colorectal cancer patients with an ostomy and provide a reference for helping these patients return to society.	277 colorectal cancer patients	15-item General Alienation Scale (GAS); 24-item Social Impact Scale (SIS)	Demographic characteristics: primary caregiver: patients whose primary caregiver was their parent reported the highest levels of subjective social isolation, followed by spouses and then children (*p* = 0.013). Having cancer: type of ostomy: subjective social isolation scores were higher in patients with temporary enterostomy than in patients with permanent enterostomy (β = 5.382, *p <* 0.001); the number of hospitalizations: patients who were hospitalized more often had higher subjective social isolation scores than those who were hospitalized less often (β = 4.465, *p <* 0.001). Health status: psychological health status: stigma was positively correlated with subjective social isolation (β = 0.843, *p <* 0.001).
White et al., 2023, USA [70]	To assess the impact of the COVID-19 pandemic on depression, anxiety, and loneliness between those with and without a history of cancer.	16,231 individuals with a history of cancer and 88,409 without a history of cancer	3-Item Loneliness Scale; the Generalized Anxiety Disorder 2-item (GAD-2); the Patient Health Questionnaire-2 item (PHQ-2)	Demographic characteristics: age was negatively correlated with loneliness (*p <* 0.05); gender and comorbidity: female patients and patients with comorbidity burden were more likely to suffer from loneliness (*p <* 0.05); race and smoking status: non-Hispanic White participants and smokers experienced a higher degree of loneliness compared to non-Hispanic Black and non-Hispanic Asian patients and never-smokers, respectively (all *p <* 0.05). Psychological factors: psychological health status: anxiety and depression were positively correlated with loneliness (*p <* 0.05).
Xia et al., 2023, China [71]	To assess the loneliness, spiritual well-being, and death perception, as well as their risk factors in urological cancer patients.	324 urological (including renal, bladder, and prostate) cancer patients and 100 healthy controls	20-item UCLA Loneliness Scale-Version 3 (UCLA-V3); 12-item Functional assessment of chronic illness therapy–spiritual well-being (FACIT-Sp); 32-item Death attitude profile-revised (DAP-R)	Demographic characteristics: marital status: married patients had lower loneliness (*t* = −2.331, *p* = 0.020). Having cancer: bladder cancer (vs prostate and renal cancer) patients had higher scores of loneliness (*t* = −3.058, *p* = 0.002); systemic treatment was independently correlated with a higher UCLA-LS score than surgery treatment, local treatment, and other treatments (*t* = −3.579, *p* < 0.001).
Zhang et al., 2022, China [72]	To explore the mediating effect of social isolation on the fear of cancer recurrence and sleep quality in convalescent breast cancer patients.	338 breast cancer patients	15-item General Alienation Scale (GAS); 12-item Fear of Progression Questionnaire-short Form (FoP-Q-SF); 18-item Pittsburgh Sleep Quality Index (PSQI)	Health status: physical health status: the total scores of subjective social isolation were positively correlated with the total scores of sleep quality (r = 0.432, *p* < 0.01); psychological health status: fear of cancer recurrence was positively correlated with subjective social isolation (r = 0.485, *p* < 0.01).
**Combined Quantitative and Qualitative Studies**				
**Authors, Year, Country** **[Citation Number]**	**Aims**	**Samples**	**Instrument Used**	**Key Findings and Classifications of Influencing Factors**
Clifton et.al, 2022, USA [73]	To assess loneliness, social isolation, and social support in older adults with cancer during the pandemic.	100 older adults with cancer	UCLA Loneliness Scale long form and UCLA Three Item Loneliness Scale; PROMIS Bank Emotional Support Short Form 4a—Version 2; PROMIS Bank Social Isolation Short Form 8a—Version 2; MOS Social Support Survey; Open-ended qualitative interviews	Demographic characteristics: gender and marital status: female patients and married patients had lower loneliness; family income was negatively correlated with loneliness; residence: higher rates of loneliness were associated with individuals living alone or with an individual other than a spouse (all *p* < 0.05). Social support and social interaction: emotional support was negatively correlated with loneliness (r = −0.40, *p* < 0.05).

**Table 3:** The possible explanations for some results.

Variable	Possible Reasons
Age	(1) Younger patients experience heightened stress related to work and personal life, which lead to social isolation.
	(2) Older patients are more prone to objective social isolation and have limited social support, which may add up to feelings of isolation over time.
Gender	(1) Compared to male patients, female patients display a greater willingness to express their emotions and have more outlets to release the negative emotion, thus making them less likely to suffer from social isolation.
	(2) Females tend to be more emotional, leading to more feelings of isolation.
Educational level	Patients with higher education levels are also more likely to accept health education and medical guidance, as well as better able to discern exaggerated information on the Internet in order to reduce misunderstanding. Furthermore, patients with higher education levels tend to have better psychological adjustment, enabling them to cope more effectively with the stress and anxiety accompanying chronic illnesses.
Type D personality	Individuals with this personality trait often exhibit heightened sensitivity to the stressors associated with cancer, leading to increased negative emotions. Furthermore, concerns about potential discrimination and prejudice due to changes in physical appearance resulting from treatment may lead patients to withdraw from their social support networks. Additionally, individuals with a Type D personality may experience discomfort and apprehension in social interactions, maintaining emotional distance from family members, medical professionals, and fellow patients. This social inhibition would hinder individuals’ access to necessary support during their periods of depression, resulting in internalized emotions and increased social isolation.
Occupational status	Working patients have greater financial capacity to handle treatment-related burdens such as expenses. Additionally, employed patients would have more opportunities for interaction with others and derive a sense of accomplishment from their work.
Family income	Patients with higher would have less economic burden. In addition, they are more likely to possess a strong support system, both in terms of emotional support from friends and family. This support can significantly ease the stress and isolation that often accompany chronic illnesses, promoting a more positive outlook on life and overall well-being. Patients with higher household incomes would experience less financial burden and not expend too much energy on reducing their economic burden.
Marital status	A spouse, being a crucial source of social support, plays an indispensable role in helping cancer patients with coping with the disease more effectively. Furthermore, spouses often assume caregiving responsibilities and provide life care, spiritual comprehension, and companionship to ensure that cancer patients perceive the necessary support, which may significantly alleviate the burden experienced by cancer patients.
Treatment-related time	(1) As treatment progresses, the level of social concern and support for cancer patients tends to decrease. This decline in support could potentially result in an increased sense of social isolation for those who receive less assistance, as they may feel abandoned or neglected.
	(2) The gradual process of adjustment and acceptance that cancer patients undergo as they come to terms with their illness. As patients become more comfortable with their condition and their functional abilities improve, they may be more willing to reintegrate into society and attempt to resume their normal lives.
Informed status of the disease	The findings indicated that the informed status of a disease had an impact on the level of social isolation, which may be attributed to the fear of disease progression and death.
Physical health status	Individuals with poor physical status often lack the necessary vitality to engage in group activities, leading them to opt to stay at home. Consequently, they result in limited opportunities for social interaction. This objective social isolation, in turn, exacerbates feelings of loneliness.
Psychological health status	Individuals with negative mental health tend to engage in fewer social activities, leading to a decreased level of social integration. Consequently, they often experience a heightened sense of isolation.
Social health status	When cancer patients become ill, they might struggle to adapt to their new patient roles while finding it challenging to promptly and correctly adjust to various previously assumed societal roles.
Coping styles	Patients who engage in negative coping such as withdrawal experience diminished interest and energy in their surroundings, leading to decreased social integration. Conversely, employing positive coping strategies like problem-solving and self-consolation enables individuals to better adapt to stress and minimizes its impact on psychological and cognitive functioning, thus improving their social skills and interpersonal relationships.
Social support and social interaction	Social support plays a crucial role in coping with the emotional challenges associated with cancer. Having someone to talk to and share their fears and concerns with can provide immense comfort and reassurance. It allows patients to express their thoughts openly without judgment or criticism, which can be incredibly therapeutic. Additionally, research suggests that strong social connections can positively influence treatment outcomes for patients. The availability of social support can help patients to establish and maintain social ties, thereby reducing psychological distress among cancer patients by providing an outlet for emotional expression and offering guidance on stress management techniques. This ultimately contributes to improving mental health outcomes and quality of life.

## Data Availability

All data used in this study were completely published online.
